# A transcriptomic atlas of mammalian olfactory mucosae reveals an evolutionary influence on food odor detection in humans

**DOI:** 10.1126/sciadv.aax0396

**Published:** 2019-07-31

**Authors:** Luis R. Saraiva, Fernando Riveros-McKay, Massimo Mezzavilla, Eman H. Abou-Moussa, Charles J. Arayata, Melanie Makhlouf, Casey Trimmer, Ximena Ibarra-Soria, Mona Khan, Laura Van Gerven, Mark Jorissen, Matthew Gibbs, Ciaran O’Flynn, Scott McGrane, Peter Mombaerts, John C. Marioni, Joel D. Mainland, Darren W. Logan

**Affiliations:** 1 Sidra Medicine, PO Box 26999, Doha, Qatar.; 2 Wellcome Sanger Institute, Wellcome Genome Campus, Hinxton, Cambridge CB10 1SD, UK.; 3 European Bioinformatics Institute (EMBL-EBI), European Molecular Biology Laboratory, Wellcome Genome Campus, Hinxton,, Cambridge CB10 1SD, UK.; 4 Monell Chemical Senses Center, Philadelphia, PA 19104, USA.; 5 Max Planck Research Unit for Neurogenetics, Max von-Laue-Strasse 4, 60438 Frankfurt, Germany.; 6 Department of ENT-HNS, UZ Leuven, Herestraat 49, 3000 Leuven, Belgium.; 7 Waltham Centre for Pet Nutrition, Leicestershire LE14 4RT, UK.; 8 CRUK Cambridge Institute, University of Cambridge, Cambridge CB2 0RE, UK; 9 Department of Neuroscience, University of Pennsylvania, Philadelphia, PA 19104, USA.

## Abstract

The mammalian olfactory system displays species-specific adaptations to different ecological niches. To investigate the evolutionary dynamics of olfactory sensory neuron (OSN) subtypes across mammalian evolution, we applied RNA sequencing of whole olfactory mucosa samples from mouse, rat, dog, marmoset, macaque, and human. We find that OSN subtypes, representative of all known mouse chemosensory receptor gene families, are present in all analyzed species. Further, we show that OSN subtypes expressing canonical olfactory receptors are distributed across a large dynamic range and that homologous subtypes can be either highly abundant across all species or species/order specific. Highly abundant mouse and human OSN subtypes detect odorants with similar sensory profiles and sense ecologically relevant odorants, such as mouse semiochemicals or human key food odorants. Together, our results allow for a better understanding of the evolution of mammalian olfaction in mammals and provide insights into the possible functions of highly abundant OSN subtypes.

## Teaser

Comparative transcriptomics of olfactory mucosae offers clues into the putative functions of highly abundant ORs in mammals.

## INTRODUCTION

Odor detection in mammals is initiated by the activation of olfactory receptors (ORs) expressed in olfactory sensory neurons (OSNs), which populate the whole olfactory mucosa (WOM) (*1*). Most mature OSNs (mOSNs) predominantly express one allele of a single OR gene (*2*, *3*). Smaller subsets of mOSNs express other families of chemosensors, such as trace amine–associated receptors (TAARs), guanylate cyclases (GCs), or members of the membrane-spanning 4-pass A (MS4As) gene family (*4*). These receptors define the molecular identity and odorant response profile of OSNs, and OSNs apply a combinatorial strategy to discriminate a number of odorants vastly greater than the number of receptors present in the genome (*2*, *5*). From a phylogenetic perspective, OR genes are divided in two classes: class I, which preferentially binds hydrophilic odorants, and class II, which tends to recognize hydrophobic odorants (*6*, *7*). The complex evolutionary dynamics of OR genes have resulted in notably different species-specific repertoires, which are presumably shaped by the chemosensory information that is required for survival in each species’ niche (*4*, *8*). While cataloging the presence of orthologous OR genes among species has provided some insight into the drivers of selection (*8*), knowing the relative abundance of each OSN subtype both within and among species may provide a better understanding of these evolutionary dynamics.

Using an RNA sequencing (RNA-seq)–based approach, we have previously profiled the complete mouse and zebrafish OSN repertoires and found that they are stratified into hundreds to thousands of functionally distinct subtypes, represented across a large dynamic range of abundance in both species (*3*, *9*, *10*). While this OSN distribution is stereotyped among genetically identical mice, it varies greatly among different strains (*11*). These distributions are largely genetically controlled and have thus likely diverged under evolutionary pressures (*11*). The abundance of OSNs expressing a given OR correlates with the total volume of corresponding glomeruli in the olfactory bulb (*12*). Increasing the number of OR-expressing OSNs in mouse lowers detection thresholds (*13*), raising the possibility that more abundant expression increases sensitivity to the receptor’s ligands, providing a mechanism for adaptation to enhance the detection of important ecological olfactory cues. Therefore, olfactory transcriptome analysis is a critical first step toward identifying the most functionally relevant among the hundreds of OSN subtypes without identified odorants. Here, we investigated the transcriptional dynamics and putative functions of the olfactory systems of six mammalian species, spanning ~95 million years of evolution.

## RESULTS

**Fig. 1:**
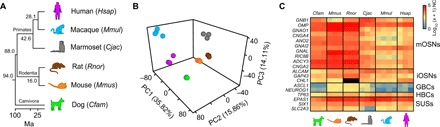
Conservation of WOM expression signatures across mammals. (**A**) RNA-seq was performed on six species, representing three mammalian lineages: Carnivora, Rodentia, and Primates. Species color scheme used is kept consistent across all figures and is as follows: light green, dog; orange, mouse; brown, rat; dark gray, marmoset; light blue, macaque; fuchsia, human. Ma, million years; *Hsap*, *H. sapiens*; *Mmul*, *M. mulatta*; *Cjac*, *C. jacchus*; *Rnor*, *R. norvegicus*; *Mmus*, *M. musculus*; *Cfam*, *C. familiaris*. (**B**) PCA of the expression levels for the 9725 “one-to-one” orthologs. Percentages of the variance explained by the PCs are indicated in parentheses. PC1 separates rodents from primates, PC2 separates Old World from New World primates and hominins, and PC3 separates dog from the other five species. (**C**) Heatmap of the expression levels of the canonical markers of the main cell populations present in the WOM samples. RNA expression levels are represented on a log_10_ scale of normalized counts (NC) plus one (0, not expressed; 5, highly expressed). There is conservation of expression among all species. mOSNs, mature OSNs; iOSNs, immature OSNs; GBCs, globose basal cells; HBCs, horizontal basal cells; SUSs, sustentacular cells. No *CHL1* ortholog was annotated in the rat genome version analyzed (black squares).

We performed RNA-seq on the WOM of male dog (*Canis familiaris*), mouse (*Mus musculus*), rat (*Rattus norvegicus*), marmoset (*Callithrix jacchus*), macaque (*Macaca mulatta*), and human (*Homo sapiens*) (Fig. 1A). We processed three biological replicates for each species, except for macaque, where we profiled four (see data file S1 for quality metrics and all gene expression data). On average, 83.85 ± 1.66% of the total reads were mapped uniquely to each corresponding genome. The intraspecific variability level among WOM replicates is extremely low (Spearman’s rho, *r*_s_ = 0.95 to 0.98; *P* < 0.0001), consistent with previous studies in laboratory animals (*9*–*11*).

To investigate the broad gene expression dynamics of the WOM across mammalian evolution, we focused on the 9725 genes that (i) have 1:1 orthology across the six species, (ii) share ≥40% amino acid identity with the human ortholog, and (iii) are expressed in at least three replicates. A principal components analysis (PCA) of the expression data revealed interspecies differentiation among subsets of orthologous genes: Principal component 1 (PC1) primarily separates rodents from primates, PC2 and PC3 separate New World monkeys (marmosets) from Old World monkeys (macaques) and hominins (humans) and dogs from all the other species, respectively (Fig. 1B and fig. S1, A and B). Hierarchical clustering (HC) analysis further supports these results (fig. S1C).

Next, we restricted our analysis to the gene markers for mOSNs, immature OSNs, horizontal basal cells, and sustentacular cells. Overall, we find that these are highly expressed across all species analyzed (Fig. 1C), consistent with previous studies (*3*, *10*). However, we do observe differences in abundance among species for some cell type–specific markers with rodents displaying the highest levels of expression for most cell type markers. The genes specific for globose basal cells (GBCs) are expressed at significantly higher levels in rodents and marmoset, when compared to other species (data file S2). Together, we have sampled, processed, and sequenced RNA of sufficiently high quality to reproducibly capture the neuronal component of WOM from inbred laboratory species (mouse and rat) and nonlaboratory species (dog, marmoset, macaque, and human).

**Fig. 2:**
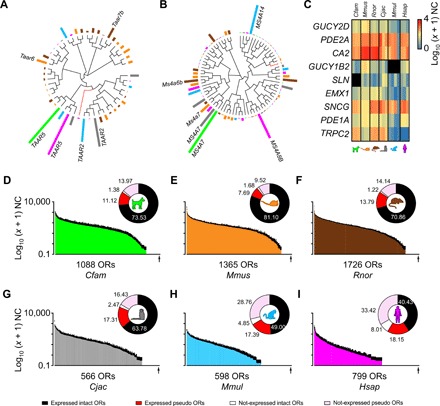
Gene expression profiles of nasal chemosensory receptors in mammals. (**A** and **B**) Unrooted phylogenetic tree and mean expression levels for all *TAAR* (A) and *MS4A* (B) receptor orthologs in the six species. Bars indicate the mean contribution (%) of each receptor to the total gene expression within each family and per species. Red branches indicate pseudo and truncated OR genes. Black branches indicate intact OR genes. (**C**) Heatmap of the expression pattern for markers of *Gucy2D^+^* (*GC-D^+^*) and *Gucy1b2^+^* OSNs in mammals. RNA expression levels are represented on a log_10_ (*x* + 1) scale of normalized counts (0, not expressed; 4, highly expressed). Black squares indicate, for a given species, genes where no orthologs were found annotated in the genome version analyzed. *TRPC2* is a pseudogene in human and macaque, and *GUCY1B2* is a pseudogene in human. (**D** to **I**) Distribution of mean expression values for each of the OR genes in the WOM of dog (D), mouse (E), rat (F), marmoset (G), macaque (H), and human (I). Genes are displayed in descending order of their mean expression values [log_10_ (*x* + 1) normalized counts]. Error bars represent the SEM from three to four individuals. Insets: circular plots show the percentages of intact and pseudo plus truncated ORs expressed (≥ 1 normalized counts) or not expressed (< 1 normalized counts) in at least one individual. Under the *x* axis, the total number of ORs within each species and the position of the last OR plotted (arrow) are noted.

Several chemosensory receptors and/or unique molecular barcodes define noncanonical (i.e., not expressing OR genes) OSN subsystems in the WOM (*4*, *14*). While orthologs for these marker genes exist in most mammals, it remains unclear whether expression is also conserved in their olfactory systems. We retrieved the annotated orthologs for these genes and analyzed their RNA-seq expression estimates. We found that the WOM of all species expresses *TAARs* and *MS4A* chemosensory receptors (Fig. 2, A and B). Within each family, the most abundant receptor and relative receptor abundance level vary greatly between species. Although *TAAR5* is the most abundantly expressed gene in human and dog, *TAAR2* is the most expressed in macaque and marmoset and *Taar6* and *Taar7b* in mouse and rat, respectively (Fig. 2A). Among the *MS4A* gene family, *MS4A8B* is the most abundant in human, *MS4A14* in macaque, *MS4A7*/*Ms4a7* in marmoset, dog, and rat, and *Ms4a6b* in mouse (Fig. 2B). Similarly, our analysis revealed that genetic markers for *GUCY2D/GC-D^+^* or *GUCY1B2^+^* OSNs are expressed in all species (Fig. 2C). The abundance of these genes varies greatly between different species with some genes being highly abundant for all species (e.g., *PDE2A* and *CA2*) and others being highly expressed only in a few species (e.g., *TRPC2*). Together, these results indicate that several specialized noncanonical olfactory subsystems are likely present across mammals, including human.

Next, we focused on OR genes, the expression of which are accurate quantitative markers for the abundance of more than a thousand OSN subtypes in mouse (*1*, *11*). We used uniquely mapped reads to quantify and analyze the distribution of OR-expressing OSN subtypes in the WOM of the six species. Because ORs can be pseudogenes in some individuals but not in others (*15*, *16*), in these analyses, we included all ORs annotated as intact and pseudogenes (hereafter referred to as ORs). In these species, the fractions of intact and class II ORs are higher than that of pseudogenes and class I OR genes, respectively (fig. S2A). The only exception is human with a higher fraction (51.56%) of pseudogenes. The OR pseudogene fraction is consistently higher than its relative contribution to the total OR gene expression pool (fig. S2A), meaning that pseudogenes are, on average, expressed at lower levels than intact genes. A similar pattern emerged when analyzing class I and class II ORs (fig. S2B). Furthermore, we found that expression estimates for ORs are more consistent between replicates from inbred strains (such as mouse and rat; *r*_s_ = 0.97 to 0.98, *P* < 0.0001) than replicates from outbred animals (such as macaque and human; *r*_s_ = 0.68 to 0.77, *P* < 0.0001; fig. S2C). Within each species, the percentage of all intact ORs expressed (≥1.0 normalized counts) in at least one replicate varies between 83.46% in human and 98.31% in rat (Fig. 2, D to I, and data file S3). In contrast, between 43.9 and 58.3% of ORs annotated as truncated or pseudogenes lack expression (<1.0 normalized counts) in any of the replicates (Fig. 2, D to I, and data file S3). With this approach, we were able to quantify a total of 323 intact human ORs (plus 145 additional truncated or pseudogenes), thus increasing the number of detected intact human ORs detected by ~18, when compared to a previous study using an array-based approach (*17*). Together, these results are indicative of the adequacy of our sampling strategy and of the quality of our WOM samples and suggest that most intact ORs are expressed, and putatively functional, in most species. We found that OR genes are expressed across a large dynamic range in all six species with only a few being highly expressed (Fig. 2, D to I). These results are consistent with previous studies in mouse and zebrafish (*3*, *9*–*11*). As OR expression correlates positively with the number of OSN subtypes in the mouse and zebrafish WOM (*10*, *11*), our results indicate that a few highly abundant OSN subtypes are present in each mammalian species with the majority present in relatively low numbers.

**Fig. 3:**
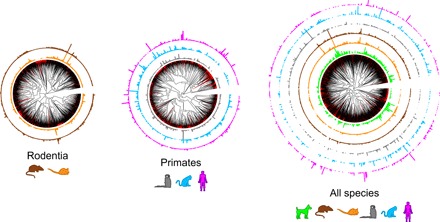
Abundance for all OR-expressing OSN subtypes across mammalian evolution. Unrooted phylogenetic trees containing the mean expression levels for all OR genes from the orders Rodentia and Primates separately and for all six species. Bars indicate the mean contribution (%) of each receptor to the total gene expression within each receptor family and per species. Red branches indicate pseudo and truncated OR genes. Black branches indicate intact OR genes.

**Fig. 4:**
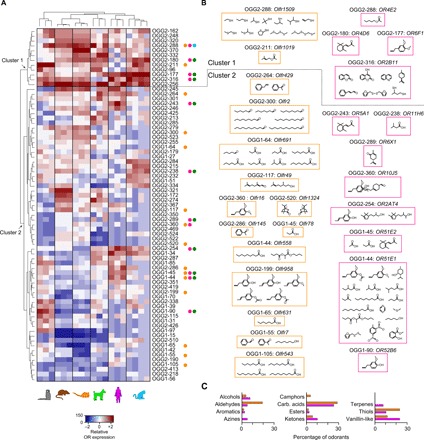
Ligand biases for highly conserved OR-expressing OSN subtypes across mammalian evolution. (**A**) Heatmap of the expression pattern for the ORs populating the highly conserved 73 OGGs across all species. Two clusters were identified (arrows): cluster 1 containing highly abundant ORs across all species and cluster 2 containing highly abundant ORs only in one or a subset of species. Normalized percentage values of expression are represented on a relative abundance scale (−2, lowly abundant; +2, highly abundant). OGGs containing human and/or mouse deorphaned ORs are indicated by orange and fuchsia circles, respectively. Mouse ORs activated by SMCs and human ORs activated by KFOs are indicated by cyan or dark green circles, respectively. Class I and class II ORs are indicated by the OGG1- and OGG2- prefixes, respectively. (**B**) Odorants recognized by the deorphaned mouse (left column and within orange rectangles) and human (right column and within fuchsia rectangles) ORs. In some, but not all cases, ligands for the same OR have similar structures. In one of three OGGs (OGG2-288) containing both deorphaned mouse and human ORs, the ligands are different in structure and perceived odors. (**C**) Percentage of odorants, according to their chemical class, activating mouse and/or human ORs.

While the overall distribution pattern of the canonical, or OR-expressing, OSN subtypes is similar among species, the relationship among them remains undetermined. Next, we plotted phylogenetic trees overlaid with the relative frequency of OSN subtypes, as defined by the abundance of the OR gene they express. To compare mean OR expression levels between individuals and species, we transformed the plotted values into the percentage of the total OR expression for each individual (data file S3). Species-specific phylogenetic trees revealed that there is no apparent clustering of the ORs that define the most abundant canonical OSN subtypes within each species (fig. S3A). However, within the order Rodentia, mouse and rat display a high level of conservation in OSN subtype frequency (Fig. 3). In contrast, when comparing abundance levels of OR-expressing OSN subtypes within the order Primates (marmoset, macaque, and human) or between any other species combinations, we observe no apparent conservation in OSN subtype representation (Fig. 3). Nonetheless, because of the high sequence similarity and large gene repertoires, it can be difficult to assign 1:1 orthology among all OR genes. To circumvent this problem, we used a classification method that sorts ORs into ortholog gene groups (OGGs) (*8*). We identified 73 OGGs showing complete 1:1 orthology (Fig. 3D). That is, each OGG is composed of a set of a single intact orthologous OR in all six species. Of these 73 OGGs, 18 are populated by class I ORs and 55 by class II ORs, hereafter referred to as OGG1- and OGG2-, respectively. HC analysis of OSN subtype abundance divided the 73 OGGs into two major clusters with all class I ORs confined to cluster 2. Half (5.67 ± 0.80) of the 12 OGGs composing cluster 1 contain highly expressed species-specific OSN subtypes (i.e., above the 90th percentile) (Fig. 4A), which is significantly above chance level for all species (binomial test, two-tail, *P* = 0.000 to 0.021; data file S4) but marmoset (binomial test, two-tail, *P* = 0.085; data file S4). In contrast, the 61 OGGs composing cluster 2 are populated by OSN subtypes that vary greatly in abundance between species and of which only ~9% are highly expressed (Fig. 4A). This is as expected by chance in all species (binomial test, two-tail, *P* = 0.077 to 0.182; data file S4) except human (binomial test, two-tail, *P* = 0.040; data file S4). Collectively, these data show that only in a few cases is phylogenetic conservation positively associated with high OR expression, suggesting that it cannot be used as a reliable predictor of highly abundant OSN subtypes in the WOM.

RNA-seq expression estimates of chemosensory receptor genes in the WOM are positively correlated with the number of OSNs expressing that given receptor (*10*, *11*). Moreover, increasing the numbers of an OSN subtype may result in increased sensitivity to the odorants that activate these cells (*13*). The interesting hypothesis is raised that for some OSN subtypes, the greater their abundance, the greater their contribution to the perception of its ligand. Under this assumption, unusually highly abundant OSN subtypes could play a role in detecting subsets of odorants that serve a critical ecological function, such as olfactory threshold or hedonics, semiochemical (SMC) detection, or food choice.

To investigate this hypothesis, we started by identifying ORs above the 90th percentile of expression in each species, hereafter also referred to as “the most” abundant OSN subtype (data file S5). By focusing our analysis in ORs with known ligands, we find that ~36% (26 of 73) of deorphaned human ORs are above the 90th percentile of expression, which is more than expected by chance (binomial test, two-tail, *P* < 0.0001). In contrast, only ~15% (13 of 89) of mouse ORs with known ligands are above the 90th percentile, as expected by chance (binomial test, two-tail, *P* = 0.1552).

We then investigated whether OSN subtypes expressing ORs in the 73 highly conserved OGGs are more likely to play a role in detecting subsets of related odorants and/or biologically relevant odors. To this end, we compiled a list of known OR-ligand pairs from literature sources (*7*, *15*, *17*–*29*) and included an OR-ligand pair based on comparable inclusion criteria from our unpublished data (fig. S4). As most OR-ligand combinations known come from studies in mouse or human, we limited all our downstream analysis to these two species (see Materials and Methods for details).

We found a total of 73 unique odorants that activate mouse and/or human ORs in 23 OGGs, of which 12 contain deorphaned mouse ORs, 8 contain deorphaned human ORs, and 3 contain both deorphaned human and mouse ORs (Fig. 4, A and B). These odorants have diverse perceived odors in humans, and on the basis of their chemical structures, we assigned them to 11 different chemical classes. In both human and mouse, carboxylic acids (cheesy/sweaty odor) are the most represented class (accounting for ~25% of all detected odorants; Fig. 4C). Thiols (sulfurous) and vanillin-like (sweet) odorants account for additional 23% in mouse and human, respectively. These two categories together represent ~44% of all detected odorants in both species. Other categories visibly different between species are aldehydes (fruity and aldehydic) in mouse and alcohols (floral and fruity) and ketones (fruity, floral, and buttery) in human. Terpenes (green and minty) and azines (animalic and pungent) were detected only by human and camphors exclusively by mouse alone. Furthermore, we found that 10 of 11 OGGs (e.g., OR5A1, OR11H6, and OR6X1) containing deorphaned human ORs that detect molecules [e.g., β-ionone, isovaleric acid, and (−)-carvone] are defined as human key food odorants (KFOs; Fig. 4, A and B, and fig. S3B), which are relevant ecological odorants (*18*). In one additional OGG, a mouse OR (*Olfr1509*) is activated by the odorant (methylthio)methanethiol (Fig. 4, A and B, and fig. S3B), which is known to be a mouse SMC (*30*). Together, these results suggest that ORs displaying high levels of 1:1 orthology across mammals play a critical olfactory role, as they are tuned to recognize specific classes of odorants, which include ecologically relevant odors.

**Fig. 5:**
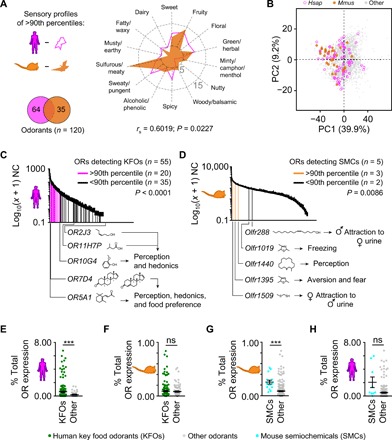
Sensory profile and detection of ecologically relevant odorants by highly abundant ORs/OSN subtypes in mouse and humans. (**A**) The sensory profiles (spider plot) of the odorants detected exclusively by either mouse or human ORs above the 90th percentile of expression (Venn diagram) are vastly overlapping and significantly positively correlated (*r*_s_ = 0.6019, *P* = 0.0227). (**B**) PCA of the 696 physicochemical descriptors of odorants detected exclusively by either mouse or human ORs above the 90th percentile of expression [see bottom left in (A)]. Percentages of the variance explained by the PCs are indicated in parentheses. The mouse- and human-specific odorants greatly overlap in the odor space. (**C** and **D**) Distribution of mean normalized counts represented on a log_10_ (*x* + 1) scale expression values for each of the OR genes in the human (C) and mouse (D) WOM. OR genes detecting at least one human KFO or a mouse SMC are indicated according to their expression percentile (fuchsia/orange, above the 90th percentile; black, below the 90th percentile). Known OR-ligand pairs playing a role in olfactory perception, hedonics, or behavior are indicated below the *x* axis. Error bars represent SEM from three sample replicates. Binomial test using Wilson-Brown method to calculate the confidence interval, two-tail. (**E** and **F**) The mean expression levels of ORs detecting human KFOs are 2.4 times higher in humans (E) but not in mouse (F). Unpaired *t* test with Welch’s correction, two-tail, ***P ≤ 0.001; ns, not significant (*P* ≥ 0.05). (**G** and **H**) The mean expression levels of ORs detecting mouse SMCs are 2.7 times higher in mouse (G) but not in humans (H). Unpaired *t* test with Welch’s correction, two-tail, ***P ≤ 0.01; ns, *P* ≥ 0.05.

The experiments above revealed a notable feature of the highly conserved and most abundant mammalian OSN subtypes and raised two important additional questions: Are highly abundant OSN subtypes within a species biased toward specific odorants? Do the most abundant OSN subtypes in different mammalian species share similar biases? To answer these questions, we first investigated possible biases in the sensory profiles (defined by their human odor qualities) and the physicochemical properties of odorants recognized by the most abundant human and mouse OSN subtypes. Unexpectedly, we found that the sensory profiles of odorants exclusively activating the mouse or human OSN subtypes above the 90th percentile of expression are highly overlapping and highly correlated (*r*_s_ = 0.6019, *P* = 0.0227; Fig. 5A). We observed only minor differences between the two species with humans preferring floral, spicy, dairy, and sweaty/pungent smells and mice showing a bias toward odorants perceived by humans as nutty, minty/camphor/menthol, and sulfurous/meaty odors. PCA of the physicochemical properties of these odorants showed similar results with all odorants intermingling in the odor space (Fig. 5B).

Next, we analyzed the expression levels of all human and mouse OSN subtypes expressing ORs that detect KFOs, SMCs, or other odorants. The analysis revealed an enrichment above the 90th percentile of expression for OSN subtypes in both human (binomial test, two-tail, *P* < 0.0001) and mouse (binomial test, two-tail, *P* = 0.0086) detecting KFOs and SMCs, respectively (Fig. 5, C and D). We found that the five human ORs known to contribute to odor perception and hedonics detect KFOs and the two (*OR7D4* and *OR5A1*) playing a role in food preferences (*15*, *24*, *25*, *31*–*33*) are among the most abundant OSN subtypes (Fig. 5C). Similarly, three mouse ORs (*Olfr1509*, *Olfr1395*, and *Olfr1440*) detecting at least one SMC (*26*–*30*) are also above the 90th percentile (Fig. 5D). OSN subtypes detecting other odorants are also enriched in human (binomial test, two-tail, *P* = 0.0086) but not in mouse (binomial test, two-tail, *P* = 0.3730) (fig. S5, A and B).

To obtain a second line of evidence supporting the putative enrichment (above the 90th percentile of expression) of OSN subtypes sensing human KFOs and mouse SMCs, we compared the mean expression values of these subtypes against the ones binding only other odorants. We found that human ORs detecting at least one KFO have on average ~2.4-fold higher expression levels than ORs detecting other odorants (unpaired *t* test with Welch’s correction, two-tail, *P* = 0.0030; Fig. 5E). Moreover, a similar analysis with mouse ORs revealed no significant differences in expression between OSN subtypes detecting human KFOs or other odorants (Fig. 5F). In line with these results, the average expression levels of mouse ORs detecting at least one SMC is ~2.7-fold higher than ORs not detecting SMCs (unpaired *t* test with Welch’s correction, two-tail, *P* = 0.0004; Fig. 5G). Furthermore, a similar analysis for human revealed no significant differences in expression between ORs detecting mouse SMCs or other odorants (Fig. 5H).

## DISCUSSION

Genes involved in sensing an animal’s immediate environment are under strong evolutionary pressure (*34*–*36*). From identifying kin, food sources, and sexually receptive mates to avoiding predation and disease, appropriate perception of environmental sensory cues is critical for survival and reproduction. The importance of sensing the chemical environment is reflected in the genetic investment in encoding ORs, which comprise the largest gene families in mammals. Since each mOSN expresses just one allele of one receptor gene (*3*, *37*), calculating total mRNA abundance of each OR in the WOM samples permitted us to assess which receptors have been favorably selected for expression in OSNs (*11*). Here, we profiled the transcriptomes of the WOM samples from six mammalian species from three different orders (Carnivora, Rodentia, and Primates), covering ~95 million years of mammalian evolution.

Overall, conservation was apparent at the whole transcriptome level and for the molecular markers for most major olfactory cell types populating the mammalian WOM. As these molecular signatures are already present in the WOM samples from zebrafish (*10*), including the most recently found subtype expressing *MS4A*, these results are consistent with an ancient origin of all mammalian OSN subtypes, arising at least 450 million years ago. We observed that different molecular markers for atypical OSN subtypes and the cell types composing the WOM occur at different abundances in different mammalian species with rodents and marmoset displaying higher levels of expression overall. The most parsimonious explanation is that the observed differences in gene expression arise from the relative differences in WOM cell type composition among species, which can be due to either biological or technical variation, or a combination of both. From a biological perspective, age and interspecific variation in specific cell type composition may explain some of the differences we observed. For example, the total numbers of OSNs across mammalian species can range from ~6 to 225 million (*38*–*41*), and some cell types are likely to be missing entirely in humans, such as those expressing TRPC2. The expression of GBC markers appears the highest in the species in which younger individuals were sampled, consistent with a role in supporting OSN turnover. Alternatively, the lack of clear boundaries between the olfactory and respiratory epithelium in some species (dog, macaque, and human) and variation in dissection technique across noses with very different morphology could also ultimately contribute to the observed differences in gene expression.

Canonical OR genes are shaped by multiple gene birth and death events, which result in species-specific repertoires with notably different numbers of intact genes, pseudogenes, and class I and class II ORs (*8*). Most intact OR genes show evidence for expression in at least one replicate, and most pseudogenes are not expressed, consistent with previous studies (*3*, *9*–*11*). We observe that the fraction of total OR gene expression originating from pseudogenes is greater for species that are outbred, such as macaque and human. This observation is consistent with greater interindividual OR genomic variation in outbred species, resulting in a higher fraction of ORs annotated as a pseudogene in the reference genome for which functional alleles are segregating in the population (*15*, *16*, *33*).

To date, the impact of evolutionary pressures on comparative global OR gene expression across different mammalian species, and hence the corresponding OSN subtype distribution (*10*, *11*), is unknown. We found that the global distribution profile of OSN subtypes expressing ORs is unexpectedly similar to the WOM samples from different mammalian species with the subtypes consistently distributed across a large dynamic range. It is unclear why, in all vertebrate species we have analyzed to date, a few OSN subtypes are present at high frequency with the majority having a low abundance. This distribution pattern may be an emergent property of the complex multistep processes that regulate OR singularity (*42*, *43*). The highly abundant OSN subtypes can be consistent between closely related species, such as in mouse and rat but not over greater evolutionary distances. We noted above that OR orthology, or the phylogenetic proximity of ORs, is generally a poor predictor of the abundance of the OSN subtypes between species. Together, these results suggest that the species specificity of the olfactory subgenome extends to its regulatory elements. ORs expressed at different levels in inbred strains of mice have greater numbers of single-nucleotide polymorphism upstream of the transcriptional start site compared to those ORs expressed at the same levels. Moreover, these variants affect transcription factor binding sites (*11*).

How does cataloging the distribution of OSNs in each species advance our understanding of the sense of smell? In humans, loss-of-function variants in the *OR5A1* gene have been shown to increase the odor threshold by about three orders of magnitude to its ligand β-ionone, suggesting that this OSN subtype is the most sensitive to this ligand (*24*). Similarly, mutations in *OR7D4* significantly contribute to specific anosmias of androstenone and androstadienone (*32*). Consistent with this, we observed that OSNs expressing *OR5A1* or *OR7D4* are particularly abundant in human WOM (on average, ranked 6th and 11th, respectively). Consequently, we propose that the more abundant an OSN subtype is, the greater its contribution to the establishment of the detection threshold and perception of its ligands. Genetic variation in other highly expressed ORs may therefore make them strong candidates for causing specific anosmias or hyposmias.

We hypothesize that OSN subtypes that are more abundant in each species may be the consequence of selection for the sensitive detection of ecologically meaningful odorants for that species. Supporting this notion, we found a significant enrichment in abundance for human OSNs that detect KFOs not observed in mouse OSNs. In contrast, we found a similar enrichment for mouse SMC in mouse OSNs, but not human OSNs, albeit with a much smaller sample size.

We propose that the overrepresentation of specific OSN subtypes is an evolutionary phenomenon: driven by direct selective pressure on genetic elements that promote monogenic OR selection because OSN abundances are, in mouse at least, largely genetically encoded (*11*). However, it remains possible that the distributions of OSN subtypes result from biases in cell survival during neurogenesis, migration, projection, or circuit integration. OSN life span can be altered by odor exposure (*44*), but when directly compared to the genetic influence, the impact of odor environment on OSN abundance is subtle (*11*). Data supporting a model, whereby an increase in an OSN subtype abundance driven by odor exposure was transgenerationally inherited in mice, have been reported (*45*). If this process scaled additively across subsequent generations, then our observations could be environmentally driven. However, these data have since been challenged on statistical grounds (*46*). Unless humans differ markedly from mice in their OSN dynamics, we consider it improbable that human KFO detection in the most abundant OSN subtypes is a physiological response to KFO exposure alone.

We cannot exclude that ascertainment biases contribute to the association between ethologically relevant odorants and high OSN abundance. For example, the list we compiled of known OR-ligand pairs originates from different studies, using different experimental conditions, and tested odorant panels that differ in composition and size. Defining OR-ligand pairs consistently in terms of magnitude of response in an in vitro system is therefore challenging. Moreover, it is possible that KFOs and SMCs were differentially enriched in odorant libraries screened against orphan ORs in humans and mice, respectively. Previous studies analyzing OR-ligand pairs from the published literature showed that KFOs have a higher probability of activating ORs than other odorants (*18*, *47*). Furthermore, in accordance with previous studies (*17*, *48*), our results suggest that ORs expressed in the most abundant OSN subtypes are more likely to be deorphaned than those expressed in the least abundant OSNs, although mouse and human ORs have been the subject of systematic deorphanization efforts (*7*, *20*). Future high-throughput studies focused on unraveling the complete ligand activation patterns of all mammalian ORs will be critical to confirming, or refuting, the conclusions from this study.

## MATERIALS AND METHODS

### Olfactory mucosae sample collection

#### Mouse (M. musculus)

RNA-seq data for 8-week-old C57Bl/6J male mouse (*n* = 3) WOM samples were retrieved from a previously published study (*9*).

#### Brown Norway rat (R. norvegicus)

Ten-week-old male (*n* = 3) animals were maintained in accordance with the U.K. Home Office regulations, under a project license approved by the Wellcome Trust Sanger Institute Animal Welfare and Ethical Review Body. The entire WOM was collected, immediately frozen, and stored at −80°C.

#### Dog (C. familiaris)

WOM samples were collected from male animals (7 to 10 years old; *n* = 3) submitted by veterinary practices to Hannover University Institute for Pathology for pathological diagnosis. Tissue was collected as soon as possible following euthanasia. The entire WOM was collected before being cut into small sections and snap-frozen on liquid nitrogen. Samples were shipped on dry ice and stored at −80°C. In all cases, owner consent for use of samples in research was obtained.

#### Primates

Male rhesus macaques (*M. mulatta*) and male common marmosets (*C. jacchus*) were kept at the German Primate Center (Göttingen, Germany). Rhesus macaque (~4.5 years old; *n* = 3) WOM samples originate from a study, which was authorized by the governmental veterinary authority [the Lower Saxony State Office for Consumer Protection and Food Safety (Niedersachsisches Landesamt for Verbraucherschutz und Lebensmittelsicherheit LAVES, ref. no. 33.9-42502-04-14/1456)] according to the regulations of the German Welfare Act (Tierschutzgesetz der Bundesrepublik Deutschland) and the European Directive 2010/63/EU on the protection of animals used for experimental and other scientific purposes. Common marmoset (~1 to 10 years old; *n* = 3) WOM samples originated from animals that were humanely euthanized because of noninfection related animal welfare reasons (e.g., trauma). The use of these samples for this study was approved by the Animal Welfare and Ethics Committee of the German Primate Center.

#### Human (H. sapiens)

Nasal mucosa/olfactory epithelium was harvested during endoscopic sinus surgery (ESS) for oncological purposes in the University Hospitals of Leuven, Belgium between April 2014 and December 2016. All included male patients (*n* = 3) had written informed consent according to the study protocol approved by the Medical Ethical Committee on Clinical Investigations at the University Hospitals of Leuven on 23 April 2014 (S5648). Included individuals underwent ESS for resection of an adenocarcinoma (stages III and IV), and during the same procedure, olfactory epithelium of the contralateral (healthy) side was harvested at the olfactory groove, followed by postoperative irradiation. After collection of the sample, the tissue was kept in RNA later and sent to the Max Planck Research Unit of Neurogenetics in Frankfurt, Germany for further analysis.

### RNA-seq of WOM

RNA from the WOMs was extracted using the RNeasy Mini/Midi Kit (QIAGEN), according to the manufacturer’s protocol. mRNA was prepared for sequencing using the TruSeq RNA sample preparation kit (Illumina) with a selected fragment size of 200 to 500 base pairs (bp). All samples were sequenced on an Illumina HiSeq 2500 to generate paired-end 100-bp sequencing reads. Libraries generated yielded an average of 49.36 ± 2.61 million (means ± SE) reads. Accession numbers can be found in data file S1.

### RNA-seq data processing and alignment

To analyze the data, we first created customized general transfer format (GTF) annotation files containing all annotated ORs for each mammalian species analyzed in this study. To create custom annotation files for the different species, we downloaded OR nucleotide sequences and the corresponding relevant whole genomes from previous studies (*8*, *49*, *50*), as described below. Genome sequences and Ensembl 79 annotations from mouse (*M. musculus*; GRCm38), macaque (*M. mulatta*; MMul_1), and marmoset (*C. jacchus*; C_jacchus3.2.1) were downloaded from Ensembl (http://ensembl.org). Genome sequences and Ensembl 54 annotations from rat (*R. norvegicus*; Rnor_4.0), dog (*C. familiaris*; CanFam2), and human (*H. sapiens*; hg18) were downloaded from the University of California, Santa Cruz Genome Bioinformatics Site (http://genome.ucsc.edu).

We generated custom annotated GTF files for use in the RNA-seq mapping pipeline by first using BLAT to map the OR sequences to their respective genome. Using a custom R script, we removed ORs that were mapped with <95% identity to the genome and removed multimapping ORs. When two ORs overlapped in the same region, the best hit was kept; if there was no best hit, then we randomly kept one OR. Using the GenomicRanges (*51*) R package, we identified overlapping annotations in the downloaded GTF files and replaced these with the OR sequences. Entries for OR sequences that did not overlap with any existing annotation were appended, thus creating a new GTF file per species.

STAR v 2.4.0i (*52*) was used to index and map reads to their respective genome using the custom annotations: outFilterType = BySJout, alignSJoverhangMin = 8, alignSJDBoverhangMin = 1, outFilterMultimapNmax = 20, alignIntronMin = 20, alignIntronMax = 1,000,000, alignMatesGapMax = 1,000,000, outFilterMismatchNoverLmax = 0.04, outFilterMismatchNmax = 999. We then performed read summarization using featureCounts (*53*). Intraspecies normalization of raw counts was performed to account for sequencing depth between samples using the DESeq2 package (*54*). On average, 83.85 ± 1.66% of the total reads were mapped uniquely to the genome.

The DESeq2 package was also used to estimate the size factors and dispersion and to generate a normalized counts matrix for the 9725 one-to-one orthologs and subsequent differential gene expression analysis. Genes were considered differentially expressed if they had an adjusted *P* value of 0.05 or less (equivalent to a false discovery rate of 5%). All results from the differential expression analyses are provided in data file S2; the columns contain the following data: *baseMean* corresponds to the mean normalized expression value for the gene across all samples; *log2FoldChange* is the fold change between the two groups tested, log_2_-transformed; *lfcSE* corresponds to the SE associated with the fold change estimation; stat is the Wald statistic; *pvalue* is the *P* value of the test; and *padj* is the *P* value after adjusting for multiple testing (Benjamini-Hochberg).

### OGG cluster assignment and regression analysis

To assess the total number of clusters among the OR genes in the 73 conserved OGGs, we performed HC by creating a dissimilarity matrix based on the normalized percentage value of the expression, assuming the total number of clusters ranging from two to eight (data file S4), using the *hclust* function implemented in R. We then compared the stability of the resulting clusters based on cluster statistics including average silhouette distance (*55*), average Pearson gamma (*56*), and within-between cluster ratio (a higher value of the former two statistics and a smaller within-between cluster ratio indicates a better fit). Two clusters were determined by balancing the performance of the cluster statistics and total number of clusters for all species. Three clusters are for only humans, and three clusters are for only mice. The R package “*fpc*” (*57*) was used to obtain the cluster statistics.

### Sequence alignment and phylogenetic reconstruction of OR repertoires

OR protein sequences were aligned using Clustal Omega with default parameters. The sequence alignment was manually edited using Mega 4 as follows: (i) Sequences were trimmed upstream of the “G” motif in EC1 (~10 amino acids upstream of the “GN” motif in TM1) and downstream of the “K” motif in IC4 (~11 amino acids downstream of the “NP” motif in TM7); (ii) positions containing alignment gaps and missing data in most sequences were eliminated. Phylogenetic trees were generated using ClustalW with 100 bootstraps. Visualization and overlay of OR gene expression data were performed using EvolView (*58*).

### Odorant information and odor descriptors

All odorant structures and associated CAS numbers were retrieved from either Sigma-Aldrich (www.sigmaaldrich.com) or PubChem (https://pubchem.ncbi.nlm.nih.gov). Odor descriptors were retrieved using The Good Scents Company database (www.thegoodscentscompany.com). A comprehensive list of the cognate mouse and human OR-ligand pairs was assembled (last update: June 2017) by combining the data from the ODORactor database (*19*) and other literature sources, representing a total of 44 different studies (*7*, *15*, *17*, *18*, *20*–*29*). Human KFOs and mouse SMCs were identified from databases and previously published studies (*15*, *17*, *18*, *22*, *25*–*33*, *59*, *60*). In addition, we found that the human OR-detecting muscone (*OR5AN1*) also recognizes the structurally related KFO β-ionone (fig. S4).

### PCA analysis with the physicochemical descriptors

We calculated 4885 physicochemical descriptors for 2662 molecules using Dragon 6.0 (Talete) software. We removed descriptors where >90% of the values were identical, where the most common value was >19× more common than the second most common value or where values were missing for any odor. The remaining 696 descriptors were then used in PCA on the 2662 compounds to plot the 113 molecules of interest in the context of the full set.

### Luciferase assay

In vitro activity of the human *OR5AN1* was measured using the Dual-Glo Luciferase Assay System (Promega). Hana3A cells were cotransfected with the OR, a short form of receptor transporter protein 1 (RTP1S), the type 2 muscarinic acetylcholine receptor (M3-R), *Renilla* luciferase driven by an SV40 promoter, and firefly luciferase driven by a cyclic adenosine 5′-monophosphate response element. Eighteen to 24 hours after transfection, ORs were treated with medium or serial dilutions of odorants spanning 1 nM to 1 mM in triplicate. Odors were first diluted to 1 M stocks in dimethyl sulfoxide and then diluted from stocks to the appropriate concentration in CD293 (Gibco). Four hours after odorant stimulation, luciferase activity was measured using the Synergy 2 (BioTek). Normalized luciferase activity was calculated by dividing firefly luciferase values by *Renilla* luciferase values for each well. Results represent mean response (for three wells) ± SEM. Responses were fit to a three-parameter sigmoidal curve.

### Statistical analysis

Statistical analyses were performed using GraphPad Prism (version 6.04), PAlaeontological STatistics (version 3.14, http://folk.uio.no/ohammer/past), and the R statistical language. Data values were standardized, and HC analysis was performed using Euclidean distances with Ward’s method. For PCA, the data matrix was standardized, and correlation matrices were used to compute the eigenvalues and eigenvectors.

## Supplementary Material

http://advances.sciencemag.org/cgi/content/full/5/7/eaax0396/DC1

Download PDF

Data file S1

Data file S2

Data file S3

Data file S4

Data file S5

## SUPPLEMENTARY MATERIALS

Supplementary material for this article is available at http://advances.sciencemag.org/cgi/content/full/5/7/eaax0396/DC1 Fig. S1. Conservation of the WOM expression signatures across mammals. Fig. S2. OR gene expression in mammals. Fig. S3. Abundance and ligand biases for highly conserved canonical/OR-expressing OSN subtypes across mammalian evolution. Fig. S4. The muscone human OR, *OR5AN1*, is also weakly activated by the KFO β-ionone. Fig. S5. Distribution of mouse and human OR genes that detect exclusively other odorants. Data file S1. Sample information, accession numbers, RNA-seq quality metrics, and gene expression estimates. Data file S2. Differential expression analysis for all pairwise comparisons between the 9785 dog, mouse, rat, marmoset, macaque, and human. Data file S3. Expression estimates for the OR repertoires of dog, mouse, rat, marmoset, macaque, and human. Data file S4. Composition of the highly conserved 73 OGGs across mammals. Data file S5. Expression estimates of the most and least abundant ORs or OSN subtypes.
