# Supplementary material for: A transcriptomic atlas of mammalian olfactory mucosae reveals an evolutionary influence on food odor detection in humans
Source: Sci Adv. 2019 Jul 31;5(7):eaax0396. doi: 10.1126/sciadv.aax0396 (PMC6669018; doi:10.1126/sciadv.aax0396)
Supplement: http://advances.sciencemag.org/cgi/content/full/5/7/eaax0396/DC1 [file supp_5_7_eaax0396__index.html]

Science Advances | Science AdvancesAAASSearchScience AdvancesMenu

## Supplementary Materials

**The PDF file includes:**

- Fig. S1. Conservation of the WOM expression signatures across mammals.
- Fig. S2. OR gene expression in mammals.
- Fig. S3. Abundance and ligand biases for highly conserved canonical/OR-expressing OSN subtypes across mammalian evolution.
- Fig. S4. The muscone human OR, *OR5AN1*, is also weakly activated by the KFO β-ionone.
- Fig. S5. Distribution of mouse and human OR genes that detect exclusively other odorants.
- Legends for data files S1 to S5

Download PDF

**Other Supplementary Material for this manuscript includes the following:**

- Data file S1 (Microsoft Excel format). Sample information, accession numbers, RNA-seq quality metrics, and gene expression estimates.
- Data file S2 (Microsoft Excel format). Differential expression analysis for all pairwise comparisons between the 9785 dog, mouse, rat, marmoset, macaque, and human.
- Data file S3 (Microsoft Excel format). Expression estimates for the OR repertoires of dog, mouse, rat, marmoset, macaque, and human.
- Data file S4 (Microsoft Excel format). Composition of the highly conserved 73 OGGs across mammals.
- Data file S5 (Microsoft Excel format). Expression estimates of the most and least abundant ORs or OSN subtypes.

**Files in this Data Supplement:**

- Adobe PDF - aax0396\_SM.pdf
