# Supplementary material for: A transcriptomic atlas of mammalian olfactory mucosae reveals an evolutionary influence on food odor detection in humans
Source: Sci Adv. 2019 Jul 31;5(7):eaax0396. doi: 10.1126/sciadv.aax0396 (PMC6669018; doi:10.1126/sciadv.aax0396)
Supplement: Download PDF [file aax0396_SM.pdf]

Science Advances
AAAS

advances.sciencemag.org/cgi/content/full/5/7/eaax0396/DC1

Supplementary Materials for

A transcriptomic atlas of mammalian olfactory mucosae reveals an evolutionary influence on food odor detection in humans

Luis R. Saraiva*, Fernando Riveros-McKay, Massimo Mezzavilla, Eman H. Abou-Moussa, Charles J. Arayata, Melanie Makhlouf, Casey Trimmer, Ximena Ibarra-Soria, Mona Khan, Laura Van Gerven, Mark Jorissen, Matthew Gibbs, Ciaran O'Flynn, Scott McGrane, Peter Mombaerts, John C. Marioni, Joel D. Mainland, Darren W. Logan*

*Corresponding author. Email: saraivalmr@gmail.com (L.R.S.); darren.logan@effem.com (D.W.L.)

Published 31 July 2019, *Sci. Adv.* **5**, eaax0396 (2019)
DOI: 10.1126/sciadv.aax0396

The PDF file includes:

Fig. S1. Conservation of the WOM expression signatures across mammals.
Fig. S2. OR gene expression in mammals.
Fig. S3. Abundance and ligand biases for highly conserved canonical/OR-expressing OSN subtypes across mammalian evolution.
Fig. S4. The muscone human OR, *OR5AN1*, is also weakly activated by the KFO β-ionone.
Fig. S5. Distribution of mouse and human OR genes that detect exclusively other odorants.
Legends of data files S1 to S5

Other Supplementary Material for this manuscript includes the following:

(available at advances.sciencemag.org/cgi/content/full/5/7/eaax0396/DC1)

Data file S1 (Microsoft Excel format). Sample information, accession numbers, RNA-seq quality metrics, and gene expression estimates.
Data file S2 (Microsoft Excel format). Differential expression analysis for all pairwise comparisons between the 9785 dog, mouse, rat, marmoset, macaque, and human.
Data file S3 (Microsoft Excel format). Expression estimates for the OR repertoires of dog, mouse, rat, marmoset, macaque, and human.
Data file S4 (Microsoft Excel format). Composition of the highly conserved 73 OGGs across mammals.
Data file S5 (Microsoft Excel format). Expression estimates of the most and least abundant ORs or OSN subtypes.

SUPPLEMENTARY MATERIAL

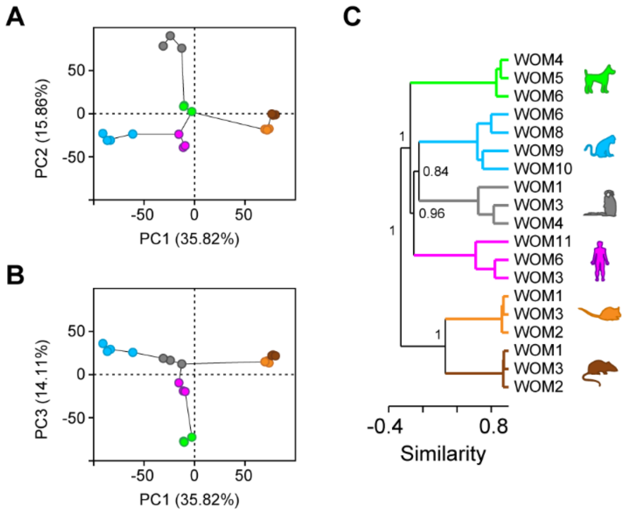

Fig. S1. Conservation of the WOM expression signatures across mammals. (**A, B**) Principal component analysis (PCA) of the expression levels for the 9725 orthologs. Percentages of the variance explained by the principal components (PCs) are indicated in parentheses. PC1 separates rodents from primates, PC2 old- from new-world primates, and PC3 separates dogs from the remaining species. (**C**) Hierarchical clustering analysis (HC) of the tissue expression profiles for the 9725 Biomart ortholog pairs. Bootstrap values (100 bootstraps, 1 represents > 0.999) for the 5 major nodes are indicated.

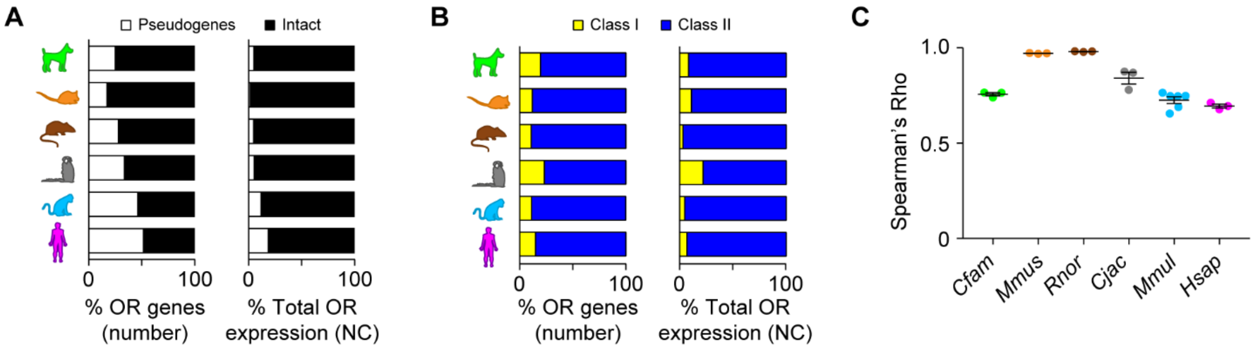

Fig. S2. OR gene expression in mammals. Percentages of Intact/Pseudogenes (**A**) and Class I/ Class II (**B**) OR genes in the genomes of the 6 analyzed mammalian species. NC – normalized counts. Note: the 'Pseudogenes' category includes both truncated and pseudogenes. (**C**) Intraspecific spearman correlation coefficients for OR repertoires are higher between replicates from inbred strains (i.e. mouse and rat), than replicates from outbred animals (i.e. dog, marmoset, macaque and human).

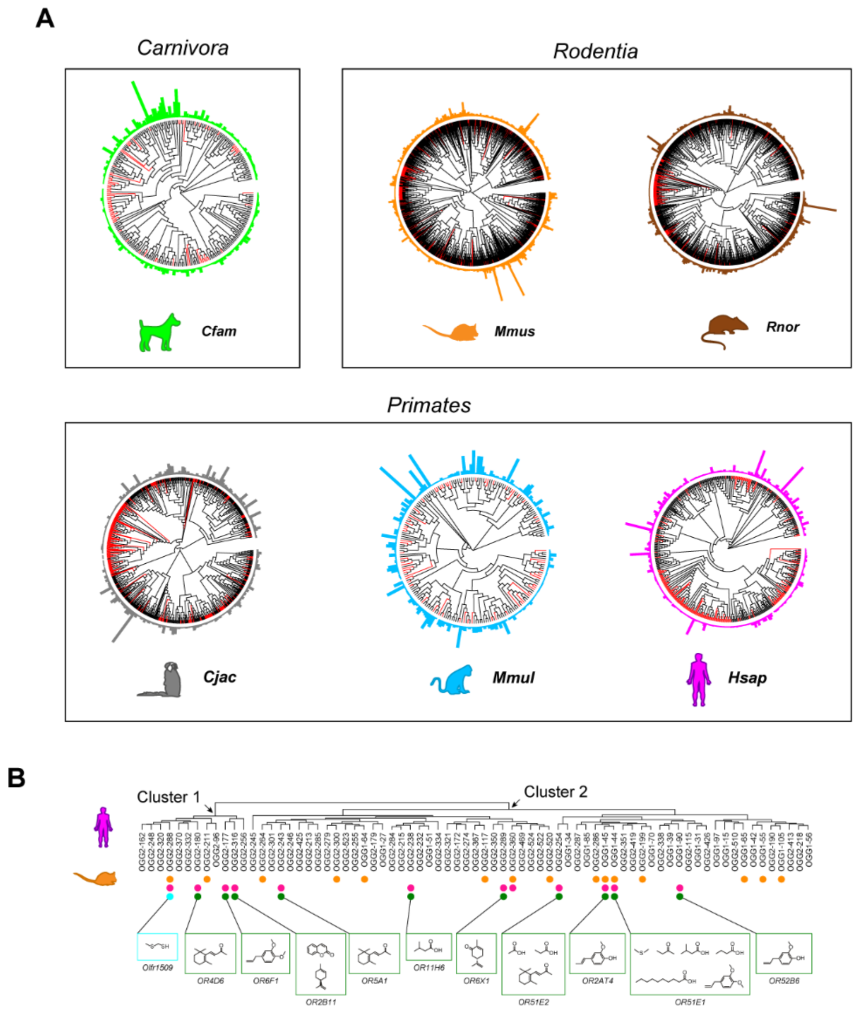

Fig. S3. Abundance and ligand biases for highly conserved canonical/OR-expressing OSN subtypes across mammalian evolution. (**A**) Unrooted phylogenetic trees containing the mean expression levels for all ORs for all six species analyzed. Bars indicate the mean contribution (%) of each receptor to the total gene expression within each receptor family, and per species. Red branches indicate pseudo and truncated OR genes. Black branches indicate intact OR genes. (**B**) Hierarchical clustering analysis of the expression pattern for the ORs populating the highly conserved 73 OGGs across all species. OGGs containing human and/or mouse deorphaned ORs are indicated by orange and fuchsia circles, respectively. Mouse or human ORs activated by

semiochemicals (SMCs) or key food odorants (KFOs) are indicated by cyan or dark-green circles, respectively.

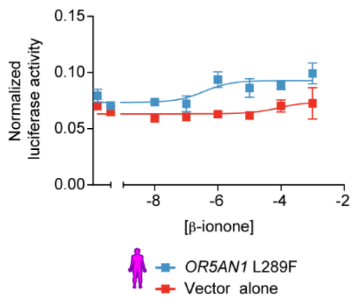

Fig. S4. The muscone human OR, *OR5AN1*, is also weakly activated by the KFO β-ionone. Hana3A cells were co-transfected with expression vectors encoding *OR5AN1* (or vector alone), a short from of receptor transporter protein 1 (RTP1S), the type 2 muscarinic acetylcholine receptor (M3-R), Renilla luciferase driven by an SV40 promoter, and firefly luciferase driven by a cyclic AMP response element. ORs were treated with medium or serial dilutions of odorants spanning 1nM to 1mM in triplicate. Odors were first diluted to 1M stocks in DMSO, then diluted from stocks to the appropriate concentration in CD293 (Gibco). Normalized luciferase activity was calculated by dividing firefly luciferase values by Renilla luciferase values for each well. Results represent mean response (for 3 wells) +/- s.e.m. Responses were fit to a three-parameter sigmoidal curve.

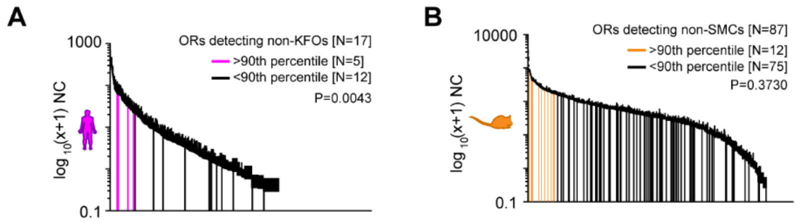

Fig. S5. Distribution of mouse and human OR genes that detect exclusively other odorants. (**A, B**) Distribution of mean normalized counts (NC) represented on a log10(x+1) scale expression values for each of the OR genes in the human (**A**) and mouse (**B**) WOM. OR genes detecting only other odorants are indicated according to their expression percentile (fuchsia/orange, above the 90th percentile; black, below the 90th percentile). Error bars represent the standard error of the mean (SEM) from 3 sample replicates. Binomial test, using Wilson/Brown method to calculate the confidence interval (CI), two-tail.

Data file S1. Sample information, accession numbers, RNA-seq quality metrics, and gene expression estimates. A dataset containing detailed information about each sample processed for RNA-seq, its ENA/EGA accession numbers, several quality control (QC) metrics and the expression values (normalized counts) for all genes in the WOM of the six analyzed mammals.

Data file S2. Differential expression analysis for all pairwise comparisons between the dog, mouse, rat, marmoset, macaque, and human. A dataset containing the expression values (normalized counts) for all genes in the WOM of the six analyzed mammals.

Data file S3. Expression estimates for the OR repertoires of dog, mouse, rat, marmoset, macaque, and human. A dataset containing the summary statistics of the ORs expressed (≥ 1 normalized count) or not expressed (< 1 normalized count) in at least 1 individual, and the expression values (normalized counts) for all ORs in the WOM of the six analyzed mammals.

Data file S4. Composition of the highly conserved 73 OGGs across mammals. A dataset containing the summary statistics, OGG composition, cluster number solution and the hypergeometric test results regarding the highly conserved 73 OGGs across all species.

Data file S5. Expression estimates of the most and least abundant ORs or OSN subtypes. A dataset containing the all ORs above the 90th- and below the 10th-percentile of expression for each analyzed species. These have also been referred throughout the text as "the most" and "the least" abundant ORs or OSN subtypes, respectively.
